# Opposing Mechanisms Involving Perceived Benefits versus Safety Partially Explained an Increase in COVID-19 Vaccination Intention among Unvaccinated Chinese Adults during a Post-Rollout Period: Results of Two Serial Surveys

**DOI:** 10.3390/vaccines9121414

**Published:** 2021-11-30

**Authors:** Yanqiu Yu, Joseph T. F. Lau, Mason M. C. Lau

**Affiliations:** Center for Health Behaviours Research, Jockey Club School of Public Health and Primary Care, The Chinese University of Hong Kong, Hong Kong, China; yuyanqiu@link.cuhk.edu.hk (Y.Y.); mason-lau@cuhk.edu.hk (M.M.C.L.)

**Keywords:** COVID-19 vaccination, behavioral intention, outcome expectancy, behavioral changes, China

## Abstract

Background: Perceptions related to COVID-19 vaccination are evolving over time, leading to potential changes in vaccination intention among unvaccinated people, which helps in the predictions of vaccination rates. This study investigated the changes in COVID-19 vaccination intention at the population level during a post-rollout period and two potential mechanisms underlying the change via the mediation/suppression effects that involve the perceived benefits/severe side effects of vaccination. Methods: Two serial random population-based telephone surveys interviewed 358 and 145 Chinese adults (aged 18–70) who were unvaccinated and who were unscheduled for COVID-19 vaccination, respectively, in May and August 2021 in Hong Kong, China. Results: The prevalence of vaccination intention increased from 14.5% to 22.8%, while the levels of perceived benefits (protection and travel-related advantages due to vaccination) and the perceived risk of severe side effects resulting from COVID-19 vaccination both significantly declined over time. Structural equation modeling found a simultaneous partial suppression effect via perceived benefits and a partial mediation effect via perceived severe side effects between the time when the surveys were conducted and COVID-19 vaccination intention, with adjustment for background factors. Conclusion: Perceptions related to COVID-19 vaccination are likely to change over time and partially account for changes in vaccination intention, sometimes in opposite directions. Ongoing health promotion may take such changes into account. Serial surveillance is warranted to monitor these changes.

## 1. Introduction

COVID-19 vaccination is seen as the most important measure for controlling the COVID-19 pandemic. A high coverage of COVID-19 vaccination in the general population is required to achieve herd immunity [1]. Reviews have identified numerous studies that have been conducted during the pre-rollout period, showing high vaccine hesitancy and low willingness, acceptance, and intention to undergo COVID-19 vaccination [2,3]. The expedite development of COVID-19 vaccines and the massive vaccination rollouts in some countries are unprecedented. As of 2 November 2021, more than 7.1 billion doses have been inoculated globally [4]. A number of countries (e.g., Canada, Singapore, and France) have achieved remarkably high vaccination coverage (>70%) in the general population within two to four months [4].

COVID-19 transmission among vaccinated people is commonly reported [5]. Although the COVID-19 vaccination rollouts in some countries have been making good progress, given the emerging uncertainties about the efficacy and protective duration of these vaccines, the herd immunity of such highly vaccinated countries cannot be guaranteed. Even within such countries/territories, the vaccine coverage in the general population is usually between 60–70% (e.g., France, Canada, and Hong Kong of China) [4], which means that about 30% or more of the population in these countries is still not being directly protected by the vaccines, making the achievement of herd immunity less feasible. Furthermore, the vaccines may be less efficacious in protecting against some of the COVID-19 variants. Ongoing health promotion efforts are thus needed for all countries, regardless of their vaccination rates. These groups of unvaccinated people may include individuals who firmly resist COVID-19 vaccination [6] and those who show psychological reactance against health promotion related to vaccination. Research in such unvaccinated groups is warranted.

It is imperative to update changes in the level and factors of vaccination intention among unvaccinated persons in post-rollout periods, as reviews show that most of the related studies on COVID-19 vaccination intention, acceptance, or hesitancy were conducted prior to the rollout [2,3]. Cognitive determinants of COVID-19 vaccination keep changing. Evidence-based information about the efficacy and safety of vaccines that strongly affects COVID-19 vaccination intention and behavior has become more available during the post-rollout periods than the pre-rollout periods [2]. However, only two identified pre-rollout studies have investigated changes in perceptions regarding COVID-19 vaccination over time (e.g., positive attitude towards COVID-19 vaccination and concerns over vaccine safety) [7,8]. No study has investigated the aforementioned changes during the post-rollout period.

There are theoretical reasons to believe that vaccination-related perceptions are prone to change over time. According to Innovation and Diffusion Theory [9], which has been applied to understand COVID-19 vaccination intention [10], the determinants for the late majority and laggards would differ from those of the innovators, early adopters, and the early majority [9]. The Theory of Planned Behavior (TPB) postulates that attitude, subjective norm, and perceived behavioral control would affect behavioral intention, which would, in turn, determine the performance of the behavior [11]. Subjective norms include descriptive norms (how many people have been vaccinated) and injunctive norms (whether a significant number of other people are supportive of vaccination); both might change over time when more people have taken up COVID-19 vaccination. The frequency of receiving cue to action (a construct of the Health Belief Model (HBM) [12]) might also have increased, as more messages about COVID-19 vaccination are appearing on the news and on social media. The HBM proposes that perceived severity and susceptibility, perceived benefits and barriers, cue to action, and self-efficacy related to health behavior are determinants for the behavior of interest [12]. Furthermore, the Social Cognitive Theory (SCT) postulates that observational learning, outcome expectancy, self-efficacy, behavioral capacity, and reinforcement can affect health behaviors [13]. In this case, observational learning and outcome expectancy might increase when more of the people in an individual’s social circle have taken up COVID-19 vaccination without experiencing severe side effects.

The present study looked at the changes in perceived benefits and perceived safety of COVID-19 vaccination, particularly during the early phase of the post-rollout period in the Hong Kong adult general population. Economists have suggested that decisions on health-related behavior largely depend on the perceived gains and losses that are associated with the behavior [14]. Additionally, the perceived benefits (e.g., protection and convenience for travelling) and the perceived side effects are amongst the strongest predictors of COVID-19 vaccination intention and behavior [15,16]. The perceived efficacy of protection is a key perceived benefit that is strongly associated with COVID-19 vaccination intention [2,3,15]. The convenience of travel, another type of perceived benefits, has provided the strongest incentive to COVID-19 vaccination among Hong Kong adults [17]. These perceptions regarding COVID-19 vaccination might have changed due to recent developments. Examples include the fast-spread threat of the mutated COVID-19 variants (e.g., the Delta variant) [18], concerns about the possible short duration of the protective effects of some vaccines [19], and the potential serious and lasting side effects of COVID-19 vaccines (e.g., Bell’s palsy) [20]. It is notable that the changes in the perceived benefits/safety might fully or partially explain (mediate) changes in COVID-19 vaccination intention over time, this has not been investigated.

Contextually, the COVID-19 vaccination policy in Hong Kong had been stable over the study period (May to August 2021). COVID-19 vaccination was free. People could choose between Pfizer-BioNTech-Fosun and Sinovac Biotech; locations and appointments were convenient; same-day or next-day vaccination was available most of the time. Regarding Pfizer-BioNTech-Fosun, the Phase III clinical trial reported 95% efficacy in protection and 66.4% efficacy in preventing severe COVID-19 symptoms [21]. Meta-analyses have indicated an average effectiveness of full vaccination against SARS-CoV-2 infection of 85–95% shortly after the completion of vaccination [22]; emerging findings have indicated satisfactory effectiveness in general [23]. Regarding Sinovac Biotech, the Phase III clinical trial reported 50.65% efficacy in protection and 83.5% efficacy against COVID-19 with at least one symptom [24]. Its effectiveness varied across countries and populations. For instance, 67% effectiveness against symptomatic COVID-19 and 85% protection against hospitalization were reported in Chile [25]. Vaccine supply is neither a determinant of COVID-19 vaccination intention and behavior nor a potential confounder of the change in vaccination intention over time in Hong Kong, as the supply has always been adequate. The governmental COVID-19 vaccination program in Hong Kong started in late February 2021. It was sluggish in the first two to three months, as the local first-dose vaccination rate was lower than 20% in May 2020; the rate, however, accelerated to 55.6% at the end of the study period (1 September 2021) and was 68.7% as of 2 November 2021 [26].

Given the background, the present study investigated (1) the difference in the prevalence of COVID-19 vaccination intention (in the next six months), comparing data obtained from two serial population-based surveys conducted about three months (Round 1) and six months (Round 2) after the beginning of the COVID-19 vaccination rollout in Hong Kong, and (2) the differences in the levels of the two types of perceptions regarding COVID-19 vaccination (perceived benefits and perceived severe side effects) over the study period. Since the direction of the changes in perceptions and intention over time were unknown, two-sided alternate hypotheses were tested. This study further tested the hypothesis that the difference in the prevalence of vaccination intention (in the next six months) between the two surveys could be explained by the level of the perceived benefits and the level of the perceived severe side effects regarding COVID-19 vaccination, i.e., the association between the survey time (Round 2 versus Round 1) and vaccination intention in the next six months would be mediated or suppressed by the perceived benefits (protection against infection among self and significant others and travel-related advantages) and the perceived severe side effects, depending on the direction of the associations between survey time and these two potential mediators.

## 2. Materials and Methods

### 2.1. Participants and Data Collection

Two serial territory-wide population-based random telephone surveys were conducted in the adult general population in Hong Kong, China. Round 1 was conducted from 14–27 May 2021, and Round 2 was conducted from 4 August to 1 September 2021. Inclusion criteria included (1) those aged 18–70 years old, and (2) those who had receive no COVID-19 vaccination nor had made an appointment to do so. Data were collected from 553 and 450 participants in the Round 1 and Round 2 surveys, respectively. Those who had either undergone COVID-19 vaccination or who had made online appointment to do so on a specific date (a maximum of three weeks after the phone survey) were excluded from the data analysis; as such, 195 surveys from Round 1 and 305 surveys from Round 2 were thus removed. The sample size that was deemed to be effective for data analysis was hence 358 and 145 for the Round 1 and Round 2 surveys, respectively.

Fixed-line telephone numbers were randomly selected from the most recently updated landline telephone directories; the last two digits were randomized to include some potentially unlisted telephone numbers. About 80.7% of the households in Hong Kong owned a landline phone in 2021 [27]. All of the telephone interviews were conducted between 6 pm to 10:30 pm to avoid over-sampling non-working individuals. Unanswered telephone calls were given at least three attempts before being classified as invalid. Unavailable eligible participants were contacted again by appointment. All of the participants were briefed by trained and experienced interviewers about the content and objectives of the study. Prospective participants were assured that participation was voluntary, refusals would have no negative consequences, and they could quit anytime without being questioned. Verbal informed consent was sought from all the participants prior to the commencement of the interview; the interviewers were required to sign a form pledging having completed the required consent procedures. The anonymous interview took about 10 to 15 min to complete. No incentives were given to the participants. The response rate (i.e., the number of completed interviews divided by the number of eligible respondents) was 56.8% and 55.7% for the Round 1 and Round 2 surveys, respectively. Ethics approval was obtained from the Survey and Behavioural Research Ethics Committee of the corresponding author’s affiliated institution (Reference No. SBRE-20-722 and No. SBRE-20-850).

### 2.2. Measures

#### 2.2.1. Background Factors

Background information was collected, including sex, age, educational level, marital status, and self-reported chronic disease status (whether the participants had at least one of the listed chronic diseases, including hypertension, diabetes, chronic pulmonary diseases, myocardial infarction, cardiac failure, cerebrovascular diseases, Alzheimer’s disease, ulcerative diseases, liver diseases, and tumors; yes/no).

#### 2.2.2. Behavioral Intention of COVID-19 Vaccination

A binary variable was created based on the 5-point response to an item that assessed the participants’ perceived chance of undergoing COVID-19 vaccination in the next 6 months. The responses were recoded into a binary variable of high intention (likely yes/definitely yes) versus low intention (half-half/likely not/definitely not).

#### 2.2.3. Perceived Benefits and Severe Side Effects of COVID-19 Vaccination

Perceived benefits involved the two domains of protective effects and travel-related advantages due to vaccination. (1) Perceived protective effect was assessed by two items regarding the level of agreement with the statements that COVID-19 vaccination could protect oneself and one’s family members/friends from contracting COVID-19 (1 = totally disagree to 5 = totally agree). The Cronbach’s alpha was 0.96 and 0.99 for the Round 1 and Round 2 surveys, respectively. (2) Travel-related advantages due to vaccination were assessed by an item about the level of agreement with the statement that COVID-19 vaccination could facilitate his/her traveling (e.g., obtaining a “vaccine passport”) (1 = totally disagree to 5 = totally agree). (3) Regarding safety, an item assessed the level of agreement with the statement that COVID-19 vaccination could induce severe side effects and even death (5 points: 1 = totally disagree to 5 = totally agree).

### 2.3. Data Analysis

The Chi-square test and the *t*-test were used to compare between-group differences (Round 2 versus Round 1) for the categorical variables and the continuous variables, respectively. The effect size of between-group differences determined by the *t*-test was indicated by Cohen’s d, where 0.2, 0.5, and 0.8 denoted a small, medium, and large effect size, respectively [28]. Associations between the background variables and COVID-19 vaccination intention were tested using univariable logistic regression analysis. Adjusted associations were tested between survey time and COVID-19-related perceptions (perceived benefits and severe side effects) and COVID-19 vaccination intention using multivariable logistic regression analysis, adjusting for the background factors (i.e., sex, age, educational level, marital status, and chronic disease status). Structural equation modeling (SEM) was conducted to test the mediation/suppression effects of the association between survey time and COVID-19 vaccination intention via the two types of perceived benefits and/or perceived severe side effects, adjusting for the background factors. As COVID-19 vaccination intention was a binary dependent variable, the Weighted Least Squared Mean and Variance (WLSMV) estimator was used [29]. The recommended criteria of model fit index included Chi-square/df ≤ 5, the Comparative Fit Index (CFI) ≥ 0.90, the Tucker–Lewis Index (TLI) ≥ 0.90, and Root Mean Square Error of Approximation (RMSEA) ≤ 0.08. By using the bootstrapping approach (*n* = 2000), an indirect effect would be considered statistically significant if its 95% confidence interval (CI) did not include zero. The mediation effect size was calculated by the proportion that the total effect was explained (mediated) by the specific path (i.e., mediation effect size equals the indirect effect of a specific path divided by the total effect) [30]. SPSS 23.0 and Mplus 7.0 were used for statistical analyses. Statistical significance was defined as the *p* value < 0.05.

## 3. Results

### 3.1. Comparing the Two Sample’s Characteristics

In Table 1, the two serial surveys did not differ significantly in terms of sex distribution (males: Round 1: 37.7%; Round 2: 33.8%; *p* = 0.409) and current marital status (married: Round 1: 62.3%; Round 2: 59.3%; *p* = 0.534). The Round 2 participants were significantly older than the Round 1 participants (Round 1: mean (SD) = 44.1 (14.0) years; Round 2: mean (SD) = 47.5 (15.3) years; *p* = 0.020)). The percentages of having participants with an educational level of college or above (Round 1: 15.9%; Round 2: 33.1%; *p* < 0.001) and chronic diseases (Round 1: 22.1%; Round 2: 31.7%; *p* = 0.023) were significantly higher in the Round 2 than they were in the Round 1 survey. All of the background factors were adjusted for in subsequent analyses.

### 3.2. Associations between Background Factors/Perceptions Related to Vaccination and Vaccination Intention

The results are presented in Table 2. The associations between the studied background factors (age, sex, educational level, marital status, and chronic disease status) and COVID-19 vaccination intention were all statistically non-significant. Adjusted for the background factors, the two types of perceived benefits were positively associated with vaccination intention (protective effect: ORa = 1.80, 95% CI: 1.27–2.55, *p* = 0.001; travel-related advantages due to vaccination: ORa =1.48, 95% CI: 1.05–2.08, *p* = 0.026), while perceived severe side effects were negatively and significantly associated with vaccination intention (ORa = 0.47, 95% CI: 0.33–0.68, *p* < 0.001) (see Table 3).

### 3.3. Testing the Differences in Two Surveys’ Prevalence of Vaccination Intention and Levels of Perceptions Related to Vaccination

In Table 1 and Table 3, it is seen that the prevalence of vaccination intention increased significantly from 14.5% during Round 1 to 22.8% during Round 2 (ORa = 1.86; 95% CI: 1.12, 3.08; *p* = 0.016). Table 4 shows that the levels of the two types of perceived benefits (perceived protective effect and perceived travel-related advantaged due to vaccination) were both significantly lower during Round 2 than they were during Round 1. The level of perceived severe side effects also declined significantly over time. The effect sizes (Cohen’s d) of the three differences over time ranged from 0.31 to 0.42 (i.e., between small and moderate).

### 3.4. Testing Mediation/Suppression between Survey Time and COVID-19 Vaccination Intention

**Figure 1:**
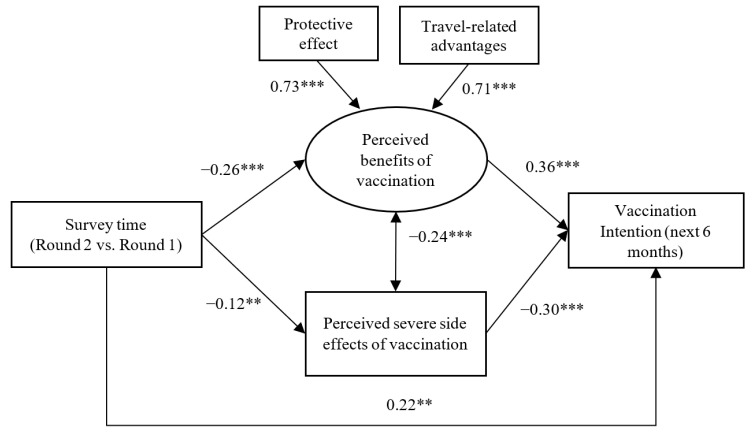
Structural equation modeling testing mediation and suppression effects between survey time and vaccination intention via perceived benefits and severe side effects of vaccination (Standardized beta coefficients were reported; **, *p* < 0.01; ***, *p* < 0.001).

Figure 1 presents the results of the SEM model testing the potential mediation/suppression mechanisms between survey time (Round 2 versus Round 1) and vaccination intention. The model showed satisfactory model fit (Chi-square/*df* = 1.31 < 5, *p* = 0.241; CFI = 0.99; TLI = 0.95; RMSEA = 0.03). The two factor loadings of the latent variable of perceived benefits were both higher than 0.70 (*p* < 0.001) and were thus satisfactory.

The mediation and suppression hypotheses were supported by the data. First, compared to the Round 1 participants, the Round 2 participants perceived lower levels of the latent variable of perceived benefits (*β* = −0.26), which were positively associated with COVID-19 vaccination intention (*β* = 0.36); an indirect suppression effect between survey time and COVID-19 vaccination intention via perceived benefits (a latent variable) was hence observed (*β* = −0.09, 95% CI: −0.14, −0.04), which was significant, as its 95% CI did not include zero. Second, the Round 2 participants tended to have lower levels of perceived severe side effects (*β* = −0.12), which was negatively associated with COVID-19 vaccination intention (*β* = −0.30); a statistically significant mediation between survey time and COVID-19 vaccination intention via perceived severe side effects was hence observed (*β* = 0.03, 95% CI: 0.01, 0.06). The suppression path via perceived benefit (a latent variable) and the mediation path via perceived safety explained 56.3% and 18.8% of the total effect, respectively. Third, a significant direct effect (*β* = 0.22) indicated higher COVID-19 vaccination intention in the Round 2 participants than it did in the Round 1 participants after considering the aforementioned mediation/suppression effects. Significant partial mediation and suppression effects were hence detected.

## 4. Discussion

This study observed a fairly low prevalence of COVID-19 vaccination intention in the next six months among unvaccinated and unscheduled adults aged 18–70 in Hong Kong (14.5% and 22.8% at about three and six months since the vaccine rollout, respectively). The latter was comparable to the 25.1% reported in another local study among unvaccinated adults who had been surveyed two months after the beginning of the rollout [17]. People with a low vaccination intention might be hesitating, waiting to see [15], or refusing COVID-19 vaccination firmly [6]. The low prevalence of COVID-19 vaccination intention is a good reminder to the government that the achievement of herd immunity through vaccination in the next six months remains an uphill mission.

The pre-rollout data recorded a relatively higher prevalence of COVID-19 vaccination intention of 37.5% to 48.7% among Hong Kong adults [31,32]. The apparent difference between pre-rollout and post-rollout prevalence may partially be explained by the exclusion of vaccinated people from this post-rollout study, as vaccinated people were more likely than unvaccinated people to indicate high vaccination intention prior to undergoing COVID-19 vaccination. Changes in related perceptions over time might also have caused the difference. Notably, the prevalence of pre-rollout COVID-19 vaccination intention seems unable to predict post-rollout vaccination intention, as a large discrepancy was detected.

It is encouraging that the prevalence of vaccination intention increased significantly from 14.5% to 22.8% in Hong Kong over the 3-month post-rollout period. The increase in intention corroborates the subsequent increase in vaccination rates that was observed after the survey period. The two surveys used identical method and measurement tools. To our knowledge, no other studies have investigated similar comparisons during the post-rollout period, while a previous local pre-rollout study found, in contrast, a decline in COVID-19 vaccination intention over time, which was explained in the report by an increase in concern over the safety of the vaccines [8,32]. The present study confirms that the levels of the perceived benefits and safety of COVID-19 vaccination continue to evolve during post-rollout period, but the direction of the change might be different from that of pre-rollout data. The set up of serial surveillance studies detecting changes that would affect the vaccination rate is warranted.

The level of the perceived efficacy of protection declined over time for reasons that have already been mentioned [33]. Breakthrough infections have commonly been reported in the mass media, and many new infections have involved who have been vaccinated against COVID-19 [34]. According to the data reported by U.S. CDC, the rate of COVID-19 infection among fully vaccinated people ranged from 5/100,000 to 130/100,000 from April to October 2021 [34]. Importantly, the participants also perceived less travel-related advantages due to COVID-19 vaccination over time. There are several plausible reasons for this. Although many countries have lifted the entry requirements for vaccinated people (e.g., the U.K., Singapore, Thailand, and the U.S), hotel quarantines of 14 to 21 days are still required upon return to Hong Kong. The resurge of COVID-19 cases in some common travel destinations (e.g., the U.K.) might also have tarnished the motivation of people in Hong Kong to travel aboard. In addition, despite lengthy negotiations and high expectations, the 21-day hotel quarantine requirement in mainland China for vaccinated Hong Kong citizens who are traveling to mainland China remains unchanged.

This study is the first one reporting a significant association between the perceived travel-related advantage and COVID-19 vaccination intention, which was stronger in this study than the widely reported association between the perceived benefits of protection and vaccination intention [31,35]. Consistently, another local study found that “vaccine passports for overseas travel” was the strongest among a list of incentives for COVID-19 vaccination (e.g., relaxation of social distancing and granting work leave to vaccinated people) [17]. The pandemic has made international travel difficult. The frequently cancelled/postponed business and recreational trips are crucial for the lifestyle of many, while the latter was positively associated with psychological wellbeing [36]. In Hong Kong, traveling to mainland China is particularly important, as the volume of daily travel across the boundary prior to the pandemic (2018) was about 314 million man-times. Notably, the change in perceived travel-related advantages and its impact on COVID-19 vaccination might vary across countries, as unlike Hong Kong (and mainland China), some countries have already loosened their inbound and outbound travel restrictions among vaccinated people. Comparative studies are warranted. Policies easing traveling arrangements among vaccinated people are likely to improve COVID-19 vaccination intention. Thus, in agreement with the socio-ecological model of health behaviors [37], structural factors such travel arrangement policies may play a strong role in affecting the COVID-19 vaccination intention among individuals.

In the present study, the level of perceived severe side effects also declined over time, which is possibly because of the wide-spread reports of low incidences of severe side effects [38]. Corroborating previous studies [15,16], perceived safety was significantly and negatively associated with COVID-19 vaccination intention. Information about vaccine safety still needs to be disseminated systematically and regularly to the general public. Overall, the moderate levels of perceived benefits and perceived severe side effects among unvaccinated people observed in this study indicate that there are substantial rooms for improvement.

Importantly, the latent variable of perceived benefits was a significant suppressor, while perceived severe side effects was a significant mediator between survey time and COVID-19 vaccination intention. The present study is possibly the first one revealing such dualistic and opposite mediation and suppression mechanisms. The increase in perceived vaccine safety over time might have pushed up the vaccination rate to some extent; the large number of people who waited to see [15,16] might feel “safe enough”. However, simultaneously, the decline in perceived COVID-19 vaccination might have pulled down the vaccination rate. The suppression effect size via perceived benefits was larger than that of the mediation path via perceived severe side effects (about 56% versus 18%). In this case, improving perceived benefits might be more effective than improving perceived safety, but that requires further proof and needs to consider country variations. Taking these two opposite mechanisms and the direct effect into account, an overall increase in vaccination intention was observed over the study period. The significant direct effect (the partial suppression and mediation effects) indicates that other potentially contradictory unmeasured suppressors and/or mediators might exist (e.g., changes in social norms and perceived risk of COVID-19 infection [39,40]).

Although the observed increase in the prevalence of vaccination intention was moderate (about 8%), the local vaccination rate increased from 55.6% at the end of the study to 68.7% as of 2 November 2021. In addition to potential changes in the perceived benefits and severe side effects, the surge might have been caused by other new anti-COVID-19 measures. For instance, tables of a larger size could be served in restaurants where the full staff were vaccinated; secondary schools can partially conduct face-to-face classes if over 70% of the teachers and students are vaccinated; some industries (e.g., healthcare workers and governmental officials) require staff to either undergo vaccination or to show proof of bi-weekly COVID-19 testing. These measures are structural factors. Again, the socio-ecological model reminds us that structural (policies and measures) and individual factors (e.g., vaccination-related perceptions) are both important in influencing COVID-19 vaccination and should be considered simultaneously in future studies. Furthermore, a number of countries/cities (e.g., the U.K. the U.S., and Hong Kong) are providing a booster to some special populations. There is currently no study looking at vaccination intention and related factors regarding the third dose. It would be interesting to understand whether the availability of a booster dose would affect the perceived benefits (and hence vaccination intention) of the first dose among unvaccinated people. Future studies are warranted.

This study has several limitations. Due to the design of a cross-sectional study, causal or temporal inferences cannot be made; longitudinal studies are warranted but lacking. Social desirability bias may exist, as COVID-19 vaccination is a prosocial behavior [41]. The characteristics of the participants and refusers may differ, although the response rate of this study (56.8%) was comparable to other local telephone surveys. Although the age distribution of the two survey samples was comparable to that of the 2019 Hong Kong census data [42], the proportion of females was slightly higher than that of the census. The age range of the sample was limited to 70 years old because of resource limitations and because older people might have different factors effecting vaccination intention; the local prevalence of vaccination in this age group was much lower than that of other age groups [26]. The sample size was smaller in Round 2 than it was Round 1, as more vaccinated participants were removed from Round 2. In the absence of available validated scales, the scales used in this study have not been validated. Some single items were used. Nevertheless, similar items have been used in previous studies on COVID-19 vaccination intention/behaviors [18,35]. Furthermore, only two major types of perceived benefits were considered in this study, while there are other perceived benefits (e.g., psychological relief). Future studies should assess comprehensive positive and negative outcome expectancies of COVID-19 vaccination. Finally, as Hong Kong has its special contexts (e.g., a good vaccine supply, specific policies, and strong need to travel to mainland China) and because the study was conducted during a specific post-rollout period, generalization to other countries and phases of roll-out need to take these aspects into account.

## 5. Conclusions

In conclusion, this study observed relatively low levels but a noticeable increase in COVID-19 vaccination intention among unvaccinated and unscheduled Hong Kong adults from Month 3 and Month 6 after the vaccination rollout began. In parallel, declines in perceived benefits (protection and travel advantages) and perceived severe side effects were observed. Dualistic mechanisms between survey time and COVID-19 vaccination intention were found to be in opposite directions, i.e., perceived benefits suppressed the association, while perceived severe side effects mediated the association. Such findings emphasize the importance of both modifying perceptions related to COVID-19 vaccination through health promotion and changing structural factors such as travel restrictions. Other potential mediators and suppressors affecting the changes in COVID-19 vaccination intention over time need to be researched, as the significant mediation/suppression effects were partial. This study reveals the dynamic nature and relationship regarding vaccination-related perceptions and intention, highlighting possible changes during the post-rollout time periods and hence the importance of monitoring through serial surveillance surveys. All in all, despite the excellent progress that has been made, there are still considerable challenges ahead before herd immunity through COVID-19 vaccination is achieved. For instance, there is no sign that the perceived benefits will increase substantially in the near future.

## Figures and Tables

**Table 1:** Comparisons of the background characteristics of the two samples.

	Round 1 n (%)	Round 2 n (%)	*p* of Chi-Square Test
**Overall**	358	145	
**Background factors**			
Sex			0.409
Females	223 (62.3)	96 (66.2)	
Males	135 (37.7)	49 (33.8)	
Educational level			<0.001
Below college	301 (84.1)	97 (66.9)	
College or above	57 (15.9)	48 (33.1)	
Marital status			0.534
Others	135 (37.7)	59 (40.7)	
Married	223 (62.3)	86 (59.3)	
Chronic disease status			0.023
No/Do not know	279 (77.9)	99 (68.3)	
Yes	79 (22.1)	46 (31.7)	
**Behavioral intention of COVID-19 vaccination in the next 6 months**			0.026
Low intention	306 (85.5)	112 (77.2)	
High intention	52 (14.5)	33 (22.8)	

**Table 2:** Background factors of behavioral intention for COVID-19 vaccination (pooled sample: *n* = 503).

	Behavioral Intention of COVID-19 Vaccination	
	ORc (95% CI)	*p*
**Age**	1.01 (0.99–1.03)	0.440
**Sex**		
Females	Ref = 1.0	
Males	0.79 (0.49–1.27)	0.335
**Educational level**		
Below college	Ref = 1.0	
College or above	1.17 (0.65–2.11)	0.610
**Marital status**		
Others	Ref = 1.0	
Married	1.08 (0.67–1.73)	0.766
**Chronic disease status**		
No/Do not know	Ref = 1.0	
Yes	0.94 (0.55–1.60)	0.809

Note: ORc = Crude odds ratio; CI = confidence interval; Ref = reference group.

**Table 3:** Factors of behavioral intention for COVID-19 vaccination (pooled sample: *n* = 503).

	Behavioral Intention of COVID-19 Vaccination	
	ORa (95% CI)	*p*
**Survey time (Round 2 versus Round 1)**	1.86 (1.12–3.08)	0.016
**Perceived benefits**		
Protective effect	1.80 (1.27–2.55)	0.001
Travel advantages	1.48 (1.05–2.08)	0.026
**Perceived severe side effects**	0.47 (0.33–0.68)	<0.001

Note: ORa = adjusted odds ratio; CI = confidence interval. The models were adjusted for background factors, including age, sex, educational level, marital status, and chronic disease status.

**Table 4:** Comparisons of perceptions related to COVID-19 vaccination assessed in the two surveys.

		Round 1	Round 2		
	Range	Mean, SD	Mean, SD	*p* of *t*-Test	Cohen’s d
**Perceived benefits**					
Protective effect	1–5	3.3, 0.9	3.0, 0.7	0.001	0.34
Travel advantages	1–5	3.6, 1.0	3.2, 1.0	<0.001	0.42
**Perceived severe side effects**	1–5	3.8, 0.9	3.5, 0.8	0.001	0.31

Note: ORa = adjusted odds ratio; CI = confidence interval. The models were adjusted for background factors, including age, sex, educational level, marital status, and chronic disease status.

## Data Availability

The data was available on reasonable request.
